# Determinants of Inadequate Health Literacy Among Faculty of Medical Technical Sciences Students in Albania: Cross-Sectional Survey

**DOI:** 10.2196/46476

**Published:** 2023-07-18

**Authors:** Etleva Rustami, Dorina Toçi, Klodiana Poshi, Elida Peka, Irida Pano, Alma Pula

**Affiliations:** 1 Department of Clinical Subject University of Medicine Tirane Tirane Albania; 2 Institution of Public Health University of Medicine in Tirane Tirane Albania

**Keywords:** Albania, health literacy, nursing, prevalence, sociodemographic factors, students, Faculty of Medical Technical Sciences, FMTS

## Abstract

**Background:**

Health literacy (HL) refers to people’s ability to find, understand, and use health information in order to make appropriate health decisions. Health literacy among students is important so that tomorrow’s health professionals can deliver high-quality health care and enhance patient education and communication.

**Objective:**

In this context, the aim of this study was to assess the HL level of Faculty of Medical Technical Sciences (FMTS) students in order to shed light on this underresearched topic in Albanian settings.

**Methods:**

A cross-sectional study involving 193 FMTS students of various study branches (nursing, midwifery, physiotherapy, and laboratory technician) was carried out during June 22-29, 2022, on the premises of the FMTS Faculty in Tirana, Albania. The international European Health Literacy Survey Questionnaire (HLS-EU-Q) standardized questionnaire, validated in Albanian, was used to collect information about FMTS students’ general HL through a face-to-face interview. Basic sociodemographic information was collected as well. Binary logistic regression was used to assess the factors associated with inadequate, problematic, or limited HL.

**Results:**

The mean level of general HL was 37.2 (on a scale from 0 [minimum HL] to 50 [maximal HL]). About one-quarter of FMTS students had inadequate (9/193, 4.7%) or problematic (38/193, 19.7%) HL, 51.3% (99/193) had sufficient HL, and 24.4% (47/193) had excellent HL. The prevalence of limited HL (inadequate and problematic HL) was higher among male than female students (6/12, 50% vs 41/181, 22.6%, respectively) and those with lower social and economic status. Upon adjusting for confounding effects, the only factor significantly increasing the likelihood of limited HL was male gender (odds ratio 8.13, 95% CI 1.68-39.39). Findings suggested that low social and economic status also increased the likelihood of limited HL, but such associations did not reach statistical significance.

**Conclusions:**

To our knowledge, this is the first study exploring the level of HL and its associated factors among FMTS students in Albania. The prevalence of limited HL was relatively high among FMTS students. There is a need for targeted interventions to increase the HL of nursing and midwifery students, such as the inclusion of HL subjects in the nursing curriculum.

## Introduction

Health literacy (HL) refers to people’s ability to find, understand, and use health information in order to make appropriate health decisions [1] in an increasingly complex health care system [2]. Despite the many types and definitions of HL (eg, one recent systematic review identified more than 250 different HL definitions) [3], virtually all experts and studies agree that poor HL is a risk factor for suboptimal health; on the other hand, HL is largely considered a modifiable risk factor, thus opening new perspectives to address illness and disease [3].

Many determinants of HL have been identified, including demographic (age, gender, education level, socioeconomic status, etc), as well as other psychosocial and cultural factors [4]. The level of individual HL at a specific time and place is determined by many factors; indeed, scientific evidence shows that there is a strong link between literacy, education, HL, and health status [5]. In this context, health literacy might be the result of a lifelong process that is built incrementally as one ages, learns (formally and informally), and interacts with the environment and other people. On the other hand, the consequences of poor HL are very diverse at the individual and community levels, and range from higher mortality and morbidity rates to higher engagement in risky health behaviors, lower health care service use at all levels, and lower participation in screening programs [4]. HL is everybody’s business because we all, at some point in our lives, will need to find, understand, and use health information and services [6].

It is obvious that HL is important for every individual and, consequently, for groups in society. This is even more critical for health professionals and especially for the young generation of students that will become the health professionals of tomorrow. This is because, in order to be able to educate patients and inform them about health risk factors, health behaviors, and their related benefits, young professionals first need to be health literate themselves [7]. Balmer and colleagues [8] argue that “the health literacy responsiveness of health organizations interacts with the health literacy of patients and their health outcomes;” in this context, a health organization first needs to have a health literate workforce in order to create health services that support the development of HL among service users (patients). As a consequence, it is important for academic institutions that train future health professionals to be aware of the health literacy level of their students, since the latter will be an additional factor in building, supporting, and developing the health literacy of the population as well [8].

Previous research in the international arena has provided some insights about HL levels and the factors associated with them, as well as HL patterns. For example, a survey among 227 nursing students (92% female) reported that HL levels were in general higher among graduate-level than entry-level students, with complex sex and age associations with various HL domains; in addition, the average HL scores were satisfactory, suggesting the appropriateness of the current university nursing curriculum without excluding the possibility for further improvement [9]. Another study among 283 nursing students (69.3% female) in Turkey investigated the factors associated with HL and reported that 29% of students had insufficient HL and 29.3% had problematic or limited HL; HL was significantly associated with family income level, type of high school graduation, and study year, whereas no significant age, sex, mother and father education, or employment status differences were detected [10]. Another survey that investigated the regional and program-level differences in HL among international nursing students concluded that the patterns of HL change are not universal but rather associated with regional program differences [11]. Even though HL and the factors associated with it are not adequately and fully explored among health professionals [12], the research among health sciences students seems promising and exciting.

In Albania, there is no information on the HL level of Faculty of Medical Technical Sciences (FMTS) students, including nursing students. In this context, we carried out an HL survey among undergraduate students of the FMTS in Albania, aiming to determine the actual HL literacy level among FMTS students and the associated factors.

## Methods

### Study Population

A cross-sectional survey was carried out among students of the FMTS in Tirana, Albania, during June 22-29, 2022. The FMTS is part of the University of Medicine, a publicly funded academic institution preparing the next generation of health professionals in Albania.

The FMTS students were recruited during the exam season of the 2021-2022 academic year. More specifically, all FMTS students of all branches (nursing, midwifery, physiotherapy, and laboratory technicians) in their first and second years of study, taking the exam on various subjects, were invited to participate in this study once they had finished their exam. The exam sessions occurred between June 20 and 29, 2022.

Of 247 students taking their exams on selected days and invited to participate, 53 students declined to do so because they did not have time, whereas 193 students agreed to participate. We did not collect any basic sociodemographic information about the nonparticipating students. Ultimately, 193 students participated in the survey, yielding a response rate of 78.1%.

### Data Collection

The international full version of the European Health Literacy Survey Questionnaire (HLS-EU-Q) instrument [13] validated in the Albanian language [14] was used to assess the health literacy level of the participants. The overall internal consistency of the instrument was high (Cronbach α=.98), and the overall test-retest reliability (stability over time) was high (Spearman coefficient ρ=0.884); in addition, the Albanian version of the HLS-EU-Q exhibited construct validity as well [14]. In line with the recommendations, the HLS-EU-Q instrument was administered face-to-face, in accordance with the instrument developer’s suggestions, using paper and pencil.

Apart from HL questions, the questionnaire included a section of basic sociodemographic information requiring the respondents to report their age, gender, branch of study, employment status, marital status, self-reported social status, and self-reported economic status.

### Statistical Analysis

The HLS-EU-Q instrument contains a total of 47 items, each one rated on a Likert scale with 4 levels (very easy, fairly easy, fairly difficult, and very difficult) [9]. The HL items’ coding was reversed in order for higher scores to indicate better HL; a general HL index was calculated, summing up all 47 items, and standardized on a scale ranging from 0 to 50, in line with the instrument developers’ recommendation [9].

General HL was categorized into the following categories: inadequate HL (score 0-25), problematic HL (>25-33), sufficient HL (>33-42), and excellent HL (>42-50) [9].

The chi-square test was used to assess the distribution of HL levels by basic sociodemographic and health behavior variables.

For our purposes, inadequate and problematic HL were collapsed into 1 single category, denoting limited HL, versus sufficient and excellent HL, which denote appropriate HL. This newly created variable was used in binary logistic regression models to assess the association of limited HL with independent sociodemographic variables. A total of 2 models of binary logistic regression were used: first, crude (unadjusted) odds ratios (OR) were reported (model 1 in binary regression analysis); subsequently, we controlled for the potential confounding effects of gender, age, branch of study, and social and economic status of the students (model 2 in binary regression analysis).

All associations with a *P* value <.05 were regarded as statistically significant. The analysis was carried out using the SPSS software, version 15 (IBM Corp).

### Ethics Approval

This study was approved by the Council of Ethics of the University of Medicine, Tirana, Albania on October 6, 2022 (decision no. 359).

Before the interview, each participating student was provided details on the purpose of the survey; subsequently, they were invited to read and sign an informed consent form. All participants provided written informed consent and were informed that their details would not be identifiable and that their anonymity would be secured. Indeed, the participating students were not required to provide their names or any other potentially identifying information. All participants provided consent before the start of the study.

## Results

The average HL score was 37.2, ranging from 16.7 to 50. Less than 1 in 20 students had inadequate HL, about 1 in 5 students had problematic HL, more than half (99/193, 51.3%) had sufficient HL, and about 1 in 4 students had excellent HL (Table 1).

The overwhelming majority of participants were female (181/193, 93.8%), more than half were in their first year of study (20 years old), and more than 90% (176/193) were studying in the nursing or midwifery branch. More than 4 in 5 students were not working (only studying) at the time of the survey; about 98% (189/193) were single; about 90% (173/193) were middle social class; and about half had average economic status (Table 2). The prevalence of inadequate and problematic HL was significantly higher among boys and students with low social and economic status, whereas no significant differences were noted with regard to the remaining sociodemographic factors (Table 2).

**Table 1:** Distribution of general health literacy score among study participants.

Variable		Value
**General health literacy score**		
	Mean (SD)	37.20 (6.69)
	Median (IQR)	37.59 (33.33-41.84)
	Minimum score	16.70
	Maximum score	50
**General health literacy level^a^, n (%)**		
	Inadequate	9 (4.7)
	Problematic	38 (19.7)
	Sufficient	99 (51.3)
	Excellent	47 (24.4)

^a^Discrepancies in the totals are due to the missing values.

**Table 2:** Distribution of health literacy levels by sociodemographic characteristics of study participants.

Variable			Total, n (%)		Health literacy, n (%)									*P* value	
					Inadequate		Problematic		Sufficient		Excellent				
**Gender**															.01^a^
	Male	12 (6.2)^b^		3 (25)^b^		3 (25)		4 (33.3)		2 (16.7)					
	Female	181 (93.8)		6 (3.3)		35 (19.3)		95 (52.5)		45 (24.9)					
**Age (years)**															.44
	20	112 (58)		3 (2.7)		24 (21.4)		58 (51.8)		27 (24.1)					
	21	81 (42)		6 (7.4)		14 (17.3)		41 (50.6)		20 (24.7)					
**Branch of study**															.46
	Nursing	97 (50.3)		4 (4.1)		17 (17.5)		51 (52.6)		25 (25.8)					
	Midwives	79 (40.9)		5 (6.3)		17 (21.5)		37 (46.8)		20 (25.3)					
	Physiotherapy	7 (3.6)		0 (0)		0 (0)		5 (71.4)		2 (28.6)					
	Laboratory technician	10 (5.2)		0 (0)		4 (40)		6 (60.6)		0 (0)					
**Employment status**															.60
	Working student	26 (13.5)		2 (7.7)		3 (11.5)		15 (57.7)		6 (23.1)					
	Not working student	167 (86.5)		7 (4.2)		35 (21)		84 (50.3)		41 (24.6)					
**Marital status**															.55
	Not married^c^	189 (97.9)		9 (4.8)		38 (20.1)		97 (51.3)		45 (23.8)					
	Married	4 (2.1)		0 (0)		0 (0)		2 (50)		2 (50)					
**Social status**															<.001
	Low	6 (3.1)		3 (50)		1 (16.7)		2 (33.3)		0 (0)					
	Middle	173 (89.6)		6 (3.5)		35 (20.2)		92 (53.2)		40 (23.1)					
	High	14 (7.3)		0 (0.)		2 (14.3)		5 (35.7)		7 (50)					
**Economic status**															.02
	Very bad or bad	3 (1.6)		1 (33.3)		1 (33.3)		1 (33.3)		0 (0)					
	Average	95 (49.2)		4 (4.2)		23 (24.2)		53 (55.8)		15 (15.8)					
	Good or very good	95 (49.2)		4 (4.2)		14 (14.7)		45 (47.4)		32 (33.7)					

^a^*P* values from the chi-square test.

^b^Absolute numbers and percentages in parentheses (row percentages for the health literacy categories, but column percentages for the totals). Discrepancies in the total numbers are due to the missing values.

^c^Single, divorced, and widowed.

Table 3 shows the sociodemographic factors associated with inadequate or problematic general HL. In multivariable adjusted analysis (model 2), the only demographic factor that statistically increased the likelihood of inadequate or problematic HL was male gender, with male students being 8.13 times more likely to report inadequate or problematic HL compared to female students (Table 3). The associations with the remaining sociodemographic factors were not significant. Nevertheless, there was a tendency for the likelihood of limited HL to be decidedly higher among students with low social status (OR 6.28 vs high social status students) and students with bad economic status (OR 3.59 vs good or very good economic status students).

**Table 3:** The association of inadequate or problematic general health literacy with basic sociodemographic factors—odds ratios (OR) from binary logistic regression.

Variable		Model 1^a^		Model 2^b^	
		OR 95% (CI)	*P* value	OR 95% (CI)	*P* value
**Gender**			.04		.01
	Male	3.42 (1.01-11.16)		8.13 (1.68-39.39)	
	Female	1.00 (reference)		1.00 (reference)	
**Age (years)**			.93		.81
	20	1.00 (reference)		1.00 (reference)	
	21	1.03 (0.53-2.01)		1.11 (0.50-2.46)	
**Branch of study**			.56 (3)^c^		.51 (3)^c^
	Nursing	1.00 (reference)	—^d^	1.00 (reference)	—
	Midwives	1.40 (0.70-2.78)	.34	1.62 (0.73-3.60)	.24
	Physiotherapy	—	—	—	—
	Laboratory technician	2.41 (0.62-9.35)	.20	2.77 (0.62-12.36)	.18
**Employment status**			.52		.44
	Working student	1.00 (reference)		1.00 (reference)	
	Not working student	1.41 (0.50-3.98)		1.57 (0.50-4.94)	
**Social status**			.07 (2)^c^		.36 (2)^c^
	Low	12.00 (1.25-115.4)	.03	6.28 (0.49-80.79)	.16
	Middle	1.86 (0.40-8.67)	.04	1.74 (0.34-8.86)	.51
	High	1.00 (reference)	—	1.00 (reference)	—
**Economic status**			.10 (2)^c^		.31 (2)^c^
	Good or very good	1.00 (reference)	.13	1.00 (reference)	.17
	Average	1.70 (0.86-3.35)	.09	1.69 (0.80-3.56)	.42
	Very bad or bad	8.56 (0.74-99.61)	—	3.59 (0.16-80.97)	—

^a^Model 1: crude (unadjusted) OR from univariate analysis.

^b^Model 2: multivariable adjusted OR controlled for gender, age, branch of study, social and economic status.

^c^Overall *P* value and degrees of freedom (in parenthesis).

^d^Not applicable.

## Discussion

### Overview

This study provided novel insights into the level and determinants of HL among students of the FMTS in Tirana, Albania. The mean HL level of FMTS students was 37.2 (on a scale from 0 to 50, where 50 indicates maximum HL), with about 1 in 4 students having inadequate or problematic HL. The prevalence of limited HL was significantly higher among boys and students with low social and economic status. On the other hand, the male gender significantly increased the likelihood of limited HL by 8.13 times.

This is among the few studies reporting on HL and its associated factors among the student population in Albania. In fact, HL among the university student population has never before been reported in Albania. Previous reports of health literacy have targeted the general population aged 18 years or older based on family physicians’ catchment areas [15], but no reports about the HL of the public university student population have been published to date. In a study conducted in a population-based sample of adult individuals aged 18 years or older in Tirana, Albania, during 2012-2014, it was reported that the average general HL level among the youngest participants (18-25 years of age) was 36.7 [15], thus being very similar to our results that reported an average HL level of 37.2 among FMTS students (20-21 years of age). Similar to the 2012-2014 study that found a significant association between HL and self-reported social and economic status, we also reported such an association among FMTS students (although the small study population size did not allow reaching statistical significance).

Our results are, in general, in accordance with the results published in the international literature. For example, this study reported that male gender is associated with an increased likelihood of reporting limited (inadequate or problematic) HL. A study among 9007 adults aged 25 years or older in Denmark reported that males were 2.3 times more likely to have inadequate HL and 1.46 times more likely to have problematic HL compared to female participants [16]. The large European HL survey, using the same HLS-EU-Q instrument, found that gender had a weak influence on general health literacy, with women tending to have higher HL than men [13], a trend that is similar to our findings. Other studies have reported that, generally, women are more likely to report adequate HL compared to men [17,18]. This can be explained by the well-established association between education and HL, implying that with the increase in education level, the HL level also increases [19,20], and the association between gender and education attainment, implying that more women than men pursue tertiary education [21-23], especially in higher nursing education [24]. As more women have higher education levels than men, women are more likely to exhibit adequate HL levels compared to men. On the other hand, the gender gap in favor of women related to adequate HL can be partially explained, besides education attainment, by the girls’ reading achievements and the boys’ disadvantages in reading comprehension, as stated by Stoet and Geary [21], who highlighted the advantages of girls and women in reading competencies compared to boys and men. This could be of particular relevance for Albania, where the PISA study, a survey of 15-year-old students that assesses the extent to which they have acquired the key knowledge and skills essential for full participation in society, revealed that the gender gap in reading (38 score points in favor of girls) was higher than the average gap in 2018 [25], and about 50% of 15-year-olds in Albania are functionally illiterate [26]. It is very likely that this gender difference will continue through the years, ultimately reflecting different levels of HL among men and women.

However, it seems that the association between HL and gender is not universal since some studies did not find a significant association between them [20,27,28], whereas some other studies have reported a reversed gender trend [29]. In this context, this study adds to the body of literature supporting a significant association between gender and HL.

The finding that lower social and economic status is associated with lower HL levels, reported in this study, is supported by the findings of the larger European HL study [13] and other studies as well [28,30-32]. In particular, it seems that financial deprivation is the most important predictor of HL level, followed by self-reported social status and education [13]. The observed association between social and economic status and HL is very likely mediated by education: in general, individuals with higher social and economic status also have better education [33], and better educated people also have better HL [34]. In this study, we could not identify a significant association between health literacy and social and economic status, but the observed trends strongly suggested the detrimental effects of lower social and economic status on health literacy.

The prevalence of adequate (sufficient or excellent) health literacy among FMTS students in this study was 75.6% (146/193). This result is very similar to another survey among 205 nursing students in Namibia, 76.5% of whom had adequate HL [35].

Although more than three-quarters of FMTS students in Albania had sufficient or excellent HL, about one-quarter of them had inadequate (9/193, 4.7%) or problematic (19.7%) HL. This finding is worrisome because today’s students are tomorrow’s nursing professionals. The HL of health care workers is critical in the context where significant associations have been reported between HL competencies and patient-centered care [36]. On the other hand, nursing professionals with adequate HL levels are of critical importance for effectively assessing and evaluating patient comprehension as well as for improving patient education and communication [37], due to their unique role and close relationship with the patients [37,38], ultimately contributing to the provision of high-quality health care, better individual and community health outcomes, and higher patient satisfaction [38]. International literature suggests the need for strategies and interventions to enhance nursing students’ HL skills not only before graduation [39] but throughout the nursing curriculum [40]. Additionally, other short interventions have proved successful in enhancing nursing students’ HL levels [41]. This is even more important in the context where the nursing discipline is the one field most lacking knowledge and awareness about the HL concept [42]. The inclusion of HL in the nursing curriculum in Albania could be of particular relevance based on the results of this study and the fact that the actual curriculum lacks such topics.

### Study Limitations

This study has several limitations. First, the relatively low response rate might have altered our findings from reality in the case that the nonparticipating students would significantly differ from those deciding to participate. Because we did not gather any information about nonparticipants, it is impossible to assess the extent and direction of this bias. Second, the relatively small study population and nonrandom selection of participants limits the generalization of the findings to all nursing student populations. Third, the cross-sectional design does not allow us to reach any definite conclusion about the temporality of events; therefore, all associations identified in this study must be interpreted with caution without implying causality.

However, the actual study has some strong points as well. For example, it is among the few studies reporting on the HL level and its determinants among nursing students in Albania. In addition, this study used an internationally validated instrument for measuring HL in Albania, thus ensuring the comparability of our results with previous national and international research. Finally, the use of robust statistical methods adds to the overall value and contribution of the actual survey.

### Conclusion

Our findings on the prevalence and factors associated with limited health literacy among FMTS students in Albania might support policy makers and education professionals in designing and implementing gender-specific interventions in order to reduce the gender gap in HL among FMTS students in Albania. On a broader perspective, such targeted interventions need to be implemented in lower education levels and extracurricular environments as well, taking into account the sociodemographic determinants of HL.

## Abbreviations

FMTS: Faculty of Medical Technical Sciences
HL: health literacy
HLS-EU-Q: European Health Literacy Survey Questionnaire

## Data Availability

The data sets generated and analyzed during this study are available from the corresponding author on reasonable request.
